# One problem is the risk of the next: a vote for early detection and preventive intervention of coexisting psychopathology

**DOI:** 10.1007/s00787-012-0302-9

**Published:** 2012-07-18

**Authors:** Aribert Rothenberger

**Affiliations:** Child and Adolescent Psychiatry, University Medicine Göttingen, von-Siebold-Str. 5, 37075 Göttingen, Germany

As the readers of this Journal know ECAP and ESCAP (European Society for Child and Adolescent Psychiatry) jointly award the “ECAP-ADHD-Paper of the Year”^1^ at a European meeting for *Child and Adolescent Psychiatry* [13]. For the winners of the year 2011, the event chosen was the Eunethydis^2^ 2nd International ADHD Conference in Barcelona (23–25 May, 2012). Two papers out of the 18 published deserved the special attention of the Jury:

Imeraj L, Antrop I, Roeyers H, Deschepper E, Bal S, Deboutte D. Diurnal variations in arousal: a naturalistic heart rate study in children with ADHD*. Eur Child Adolesc Psychiatry* (2011) 20:381–392

Kröger A, Hänig S, Seitz C, Palmason H, Meyer J, Freitag CM. Risk factors of autistic symptoms in children with ADHD. *Eur Child Adolesc Psychiatry* (2011) 20:561–570

Both studies reported of behavioural risk factors which might trigger problem behaviour in a kind of domino-effect. The work of Imeraj et al. [6] faces two neglected but practically important issues on ADHD, namely, circadiane sleep-wake behaviour and autonomic arousal. Both are basic subcortical functions which may influence higher order cognitive abilities and thus confound many studies on attention in ADHD. The presented data on 24-h heart-rate, reflecting an autonomic imbalance in ADHD, might be considered also in the light of the discussion about cardiovascular side effects of medication in ADHD. Especially, if it is true that already before the long-term use of stimulants, the overall heart-rate levels are increased in children with ADHD (for critical evaluation see also European Guidelines like [1, 5, 15]. Hence, early diagnostics and careful monitoring seems necessary. Kröger et al. [8] focus on the daily clinical issue of differential diagnosis and/or comorbidity of ADHD and increased autistic symptoms. Also, these subclinical symptoms may be confounders in research. Hence, Kröger et al. described risk factors of these increased autistic symptoms in ADHD. Their findings are indicative of possible genetic (maternal autistic traits) as well as environmental factors. The relevance of psychosocial risk factors (e.g. intra-familial discord, abnormal parenting, isolated family) was shown for the first time in this respect and suggests for the future to combine genetic and clinical research more directly in one study, but also to psychosocially support these families very early in their problem solving approaches.

Three original articles of this ECAP issue underline the view of clinical risk factors as triggers for further psychopathological signs.

For example, children of parents with chronic medical condition displayed a slightly increased risk for internalizing problems [14]. In the Dutch normative sample of the YSR, only 8 % of girls and 9 % of boys were categorized as clinical cases, while in this sample of 160 adolescents 12 % of girls and 11.7 % of boys were found. Although the evaluated anxious/depressed scale of the YSR predicts a DSM-IV disorder only moderately, preventive activities seem to be worthy since ongoing stress and thus chronic elevation of cortisol [3] by thoughts of loss, bereavement and unpredictability of parental health may lead to greater emotional problems. Fortunately, the adolescents of this sample displayed few externalizing problems. Here as well as in the study on ADHD and autism of Kröger et al. [8] the well-known negative impact of psychosocial risks was displayed. On the other hand, there exists evidence that a first psychopathological condition of an individual may form the risk for the appearance and/or severity of a next one.

In a large European school survey, based on 45.086 16-year-old adolescents from 16 countries, substance use (tranquillizer, tobacco, alcohol, cannabis) was found as risk factor for self-reported suicide attempts [7]. Remarkably, “the odds ratio of reporting a suicide attempt approximately doubled for every additional substance use”. The authors should be supported when they say, that we, e.g. mental health professionals and public health decision makers, need to shape existing prevention policies. As with most of the adolescents suffering from internalizing problems they are often neglected because of the demanding externalizing problems of their classmates. We need to better listen to the silence.

Finally, the complex neuropsychiatric disorder of Tourette syndrome (see also the European Guidelines in this Journal: [2, 10, 12, 16]) reflects some risks within itself, since its comorbidity-profile (especially while changing during the course of illness) makes it a challenging case for the patient and the physician [4, 11, 17].

Lebowitz et al. [9] clarify that chronic tic disorders (CTD) with coexisting obsessive–compulsive disorder is a more severe subtype of CTD, characterized not only by more severe tics but also by e.g. poorer global functioning. Further, similar to the situation of additional substance use [7], Wanderer et al. [17] found in CTD that the presence of comorbid OCD was associated with higher rates of additional comorbid diagnoses; i.e. the earlier the risky obsessive–compulsive traits can be detected in CTD and treated, the better is the precondition for the future course of CTD and patient’s psychosocial functioning/development.

In sum, our view on risk factors should not be restricted to environmental or genetic factors. Moreover, the risks within the complex, usually comorbid problem situation of a child psychiatric disorder deserves greater attention in order to prevent exacerbation while detecting and treating the risks quite early in time. In the long run, the effort would be paralleled by a positive cost-benefit-ratio for both, the patients and the society.

## Footnotes

1 Sponsored by Shire-Germany.

2 European Network on Hyperkinetic Disorders.

## References

[1] Banaschewski T, Coghill D, Santosh P, Zuddas A, Asherson P, Buitelaar J, Danckaerts M, Döpfner M, Faraone SV, Rothenberger A, Sergeant J, Steinhausen HC, Sonuga-Barke EJ, Taylor E (2006). Long-acting medications for the hyperkinetic disorders: a systematic review and European guideline. Eur Child Adolesc Psychiatry.

[2] Cath DC, Hedderly T, Ludolph AG, Stern JS, Murphy T, Hartmann A, Czernecki V, Robertson MM, Martino D, Munchau A, Rizzo R, ESSTS Guidelines Group (2011). European clinical guidelines for Tourette syndrome and other tic disorders. Part I: assessment. Eur Child Adolesc Psychiatry.

[3] Christiansen H, Oades RD, Psychogiou L, Hauffa BP, Sonuga-Barke EJ (2010). Does the cortisol response to stress mediate the link between expressed emotion and oppositional behavior in attention-deficit/hyperactivity-disorder (ADHD)?. Behav Brain Funct.

[4] Freeman R and the Tourette Syndrome International Database Consortium (2007) Tic disorders and ADHD—answers from a world-wide clinical data set on Tourette Syndrome. Eur Child Adolesc Psychiatry 16(suppl 1):i15–i23 10.1007/s00787-007-1003-7 17665279

[5] Graham J, Banaschewski T, Buitelaar J, Coghill D, Danckaerts M, Dittmann RW, Döpfner M, Hamilton R, Hollis C, Holtmann M, Hulpke-Wette M, Lecendreux M, Rosenthal E, Rothenberger A, Santosh P, Sergeant J, Simonoff E, Sonuga-Barke E, Wong IC, Zuddas A, Steinhausen HC, Taylor E, for the European Guidelines Group (2011). European guidelines on managing adverse effects of medication for ADHD. Eur Child Adolesc Psychiatry.

[6] Imeraj L, Antrop I, Roeyers H, Deschepper E, Bal S, Deboutte D (2011). Diurnal variations in arousal: a naturalistic heart rate study in children with ADHD. Eur Child Adolesc Psychiatry.

[7] Kokkevi A, Richardson C, Olszewski D, Matias J, Monshouwer K, Bjarnason T (2012) Multiple substance use and self-reported suicide attempts by adolescents in 16 European countries. Eur Child Adolesc Psychiatry. doi:10.1007/s00787-012-0276-7 10.1007/s00787-012-0276-7 22535305

[8] Kröger A, Hönig S, Seitz C, Palmason H, Meyer J, Freitag CM (2011). Risk factors of autistic symptoms in children with ADHD. Eur Child Adolesc Psychiatry.

[9] Lebowitz ER, Motlagh MG, Katsovich L, King RA, Lombroso PJ, Grantz H, Lin H, Bentley MJ, Gilbert DL, Singer HS, Coffey BJ, the Tourette Syndrome Study Group, Kurlan MR; Leckman JF (2012) Tourette syndrome in youth with and without obsessive compulsive disorder and attention deficit hyperactivity disorder. Eur Child Adolesc Psychiatry. doi:10.1007/s00787-012-0278-5 10.1007/s00787-012-0278-5 PMC3722559 22543961

[10] Müller-Vahl KR, Cath DC, Cavanna AE, Dehning S, Porta M, Robertson MM, Visser-Vandewalle V, ESSTS Guidelines Group (2011). European clinical guidelines for Tourette syndrome and other tic disorders. Part IV: deep brain stimulation. Eur Child Adolesc Psychiatry.

[11] Roessner V, Becker A, Freeman R, Rothenberger A, the Tourette Syndrome International Database Consortium (2007). Developmental psychopathology concerning the association of tic disorders and ADHD. Eur Child Adolesc Psychiatry.

[12] Roessner V, Plessen KJ, Rothenberger A, Ludolph AG, Rizzo R, Skov L, Strand G, Stern JS, Termine C, Hoekstra PJ, ESSTS Guidelines Group (2011). European clinical guidelines for Tourette syndrome and other tic disorders. Part II: pharmacological treatment. Eur Child Adolesc Psychiatry.

[13] Rothenberger A (2011). Supporting researchers: mentoring and awards of the journal. Eur Child Adolesc Psychiatry.

[14] Sieh DS, Visser-Meily JMA, Oort FJ, Meijer AM (2012) Risk factors for problem behavior in adolescents of parents with a chronic medical condition. Eur Child Adolesc Psychiatry. doi:10.1007/s00787-012-0279-4 10.1007/s00787-012-0279-4 PMC3411286 22543962

[15] Taylor E, Doepfner M, Sergeant J, Asherson P, Banaschewski T, Buitelaar J, Coghill D, Danckaerts M, Rothenberger A, Sonuga Barke E, Steinhausen HC, Zuddas A (2004). European clinical guidelines for hyperkinetic disorder—first update. Eur Child Adolesc Psychiatry.

[16] Verdellen C, van de Griendt J, Hartmann A, Murphy T, ESSTS Guidelines Group (2011). European clinical guidelines for Tourette syndrome and other tic disorders. Part III: behavioural and psychosocial interventions. Eur Child Adolesc Psychiatry.

[17] Wanderer S, Roessner V, Freeman R, Bock N, Rothenberger A, Becker A (2012). Relationship of obsessive-compulsive disorder to age-related comorbidity in children and adolescents with Tourette syndrome. J Dev Behav Pediat.

